# The IGF signalling pathway in Wilms tumours - A report from the ENCCA Renal Tumours Biology-driven drug development workshop

**DOI:** 10.18632/oncotarget.2485

**Published:** 2014-09-16

**Authors:** Mariana Maschietto, Jocelyn Charlton, Daniela Perotti, Paolo Radice, James I Geller, Kathy Pritchard-Jones, Mark Weeks

**Affiliations:** ^1^ Cancer Section, Institute of Child Health, University College London, London WC1N 1EH, UK; ^2^ Molecular Bases of Genetic Risk and Genetic Testing Unit, Department of Preventive and Predictive Medicine, Fondazione IRCCS Istituto Nazionale dei Tumori, Milan, Italy; ^3^ UC department of paediatrics, Cincinnati Children's Hospital, Cincinnati, Ohio, USA

**Keywords:** IGF2, IGF signalling pathway, Wilms tumour, targeted therapy, DNA methylation

## Abstract

It is hypothesised that Wilms tumour (WT) results from aberrant renal development due to its embryonic morphology, associated undifferentiated precursor lesions (termed nephrogenic rests) and embryonic kidney-like chromatin and gene expression profiles. From the study of overgrowth syndrome-associated WT, germline dysregulation was identified in the imprinted region at 11p15 affecting imprinted genes *IGF2* and *H19*. This is also detected in ~70% sporadic cases, making this the most common somatic molecular aberration in WT. This review summarises the critical discussion at an international workshop held under the auspices of The European Network for Cancer Research in Children and Adolescents (ENCCA) consortium, where the potential for drug development to target *IGF2* and the WT epigenome was debated. Here, we consider current cancer treatments which include targeting the IGF pathway and the use of methylation agents alone or in combination with other drugs in clinical trials of paediatric cancers. Finally, we discuss the possibility of the use of these drugs to treat patients with WT.

## INTRODUCTION

Wilms tumour (WT) or nephroblastoma is the most frequent renal tumor of childhood affecting one in 10,000 children, with a peak incidence between two and three years of age [1]. With an overall cure rate ≥85%, WT is one of the successes of pediatric oncology. In the Western world, the majority of patients affected by WT are treated within trials conducted by the International Society of Pediatric Oncology (SIOP, Europe) or the Children's Oncology Group (COG, formerly NWTSG, North America) [2]. WTs are frequently large and exhibit radiological, macroscopic and histological heterogeneity. At present, there is evidence of at least three pathways that are associated with WT development: the WNT/β-catenin pathway (15-20% of non-anaplastic WTs), the IGF2 pathway (69% of all WTs) and the p53 pathway (anaplastic WTs, 5-10% of all WTs). At least in part, these may be responsible for the heterogeneity in clinical phenotype and outcome observed in WTs.

Under the auspices of the European Network for Cancer Research in Children and Adolescents (ENCCA) consortium, a European Union Framework 7-funded program (http://www.encca.eu/Pages/home.aspx) a ‘Biology Driven Drug Development Renal Tumors Workshop’ was convened to review the evidence for the genetic pathways disrupted in WT and to develop a translational research strategy. This workshop was part of a wider strategic initiative to involve international experts in the biology of childhood cancers, drug development and early phase clinical trials in children and relevant adult diseases, to accelerate biology-driven drug development and clinical trials across Europe. The outcome of this workshop is being published as two focused reviews. The first discussed the significance of the canonical WNT signaling pathway in a subset of WTs and related therapeutic opportunities [3]. Here, we discuss the subset of WTs with epigenetic aberrations at 11p15 resulting in activation of the IGF signaling pathway and the therapeutic opportunities provided by targeting either the IGF pathway or the epigenome. The IGF pathway is comprised of a complex network of molecules stimulated by insulin-like growth factors (IGFs), which are synthesized by almost any tissue in the body and are important mediators of growth, development, and survival including the embryonic kidney [4]. There are an increasing number of studies highlighting their role in establishing a transformed phenotype in an increasing number of malignancies.

### Wilms tumours arise from aberrant renal development and show epigenetic features of embryonic kidney

WT is an embryonic tumour that morphologically resembles foetal kidney with varying proportions of blastemal, epithelial and stromal cells. Immature tubular structures are observed and in some cases skeletal, fat and muscle tissue suggesting a multipotent cell of origin [5]. Concurrent with its embryonal morphology, gene expression profiling has established that WTs express genes corresponding to the earliest stage of metanephric development and under-express genes from later stages of renal development [6]. Furthermore, the cellular composition of WT shows retention of expression patterns reflecting embryonic counterparts [7] Evidence has shown that depending on the location of expression of key renal development gene CITED1, WT cell lines obtain either stem-like properties (cytoplasmic expression, as seen in normal development) or tumourigenic properties (nuclear expression, as seen in WT) [8].

More recent studies have analysed the WT epigenome, which encompasses features that effect gene expression without altering the genetic coding sequence, and found similar results. Epigenetic mechanisms can be broadly classified into chromatin remodelling (by covalent histone modification or protein binding) and DNA cytosine methylation. In detail, researchers identified regions of open chromatin that permit active gene expression (with histone 3 lysine 4 trimethylation [H3K4me3]) and regions of closed chromatin that prevent gene expression (with H3K27me3) in WT, embryonic kidney (EK), embryonic stem cells (ESCs) and normal kidney (NK).

Comparisons between tissues identified open chromatin structures in WT but not NK at genes involved in kidney and mesoderm development which are also highly expressed in foetal kidney. In addition, identification of a network of genes that showed the same “bivalent” chromatin structure (both active and repressive marks simultaneously) and low expression in both WT and ESCs, but were actively expressed in NK, led to the conclusion that developmental arrest occurs in undifferentiated metanephric mesenchyme as the genes required for differentiation (*FOXD1* and *LHX1* which are involved in stromal and epithelial differentiation, respectively) remained in a poised state awaiting differentiation signals [9]. This evidence suggests that retaining epigenetic features of early renal development is important in the early stages of disease.

Supporting this theory, aberrant epigenetic events have been considered as the earliest events in tumourigenesis whereby epigenetic disruption results in a pool of tumour-progenitor cells. Within these cells, gene-specific epimutations can occur resulting in cellular transformation [10, 11]. Tumours later acquire both epigenetic and genetic plasticity that is proposed to lead to tumour heterogeneity [12]. Therefore, whilst during normal development, epigenetic modifications are remodelled to define embryo patterning and for organ and cell type specification and then upon terminal differentiation is maintained to sustain cell identity, when disrupted (during development or somatically), the epigenome may play a role in cancer initiation and progression, giving the same effect as a “classical” DNA mutation.

### Epigenetics of Wilms tumour

Aside from the developmental epigenetic features observed in WT, further aberrant epigenetic events have been observed that are analogous to this “classical” DNA mutation (summarised in Table 1). These occur by aberrant site-specific or global changes in DNA CpG methylation or chromatin structure. In detail, CpG sites are regions of DNA where a cytosine is located next to a guanine nucleotide. In general, gain of DNA methylation at CpG residues can result from the overexpression of DNA (cytosine-5)-methyltransferase 1 (*DNMT1*) and DNA (cytosine-5)-methyltransferase 3 beta (*DNMT3b*), which are regulated by *TP53* [13, 14]. As well as increase in DNA methylation, trimethylation of histone 3 (H3) at lysine (K) 27 (H3K27me3) also causes gene repression by promoting a closed chromatin structure. Alternatively, loss of DNA methylation, trimethylation of H3K4 or K36, monomethylation of H3K4 or acetylation of H3K36 promote an open chromatin structure, and the binding of transcription factors [9, 15-19]. In cancer, these changes in DNA methylation and chromatin accessibility are associated with the silencing or the overexpression of tumour suppressor genes and oncogenes, respectively (reviewed in [20]).

Of the known epimutations in WT, epigenetic aberration at 11p15 has received the most attention due to its association with Beckwith-Wiedemann Syndrome (BWS), a paediatric overgrowth disorder with germline gain of methylation at 11p15 and functional relationship with expression of imprinted genes *IGF2* and *H19* [21, 22]. There are over 40 human imprinted genes that show parental allele-specific expression [23]. This monoallelic expression tightly controls the levels of the proteins encoded by imprinted genes, usually important factors of embryonic growth, placental growth or adult metabolism [24]. The regulation of imprinted genes is largely dependent on DNA methylation marks, which are laid down during embryological development of germ cells. Once in place, the methylation status of precise chromosomal regions, imprinting control regions (ICRs), is read by either of two mechanisms, chromatin barrier formation or untranslated RNAs, thereby ensuring that only the maternal or paternal allele is expressed [25, 26]. Each imprinted gene is classified as maternal or paternal according to the expressed allele. Misregulation of imprinted gene expression (loss of imprinting [LOI]) is seen frequently in a large variety of human tumours [27]. Specifically, LOI of *IGF2* and *H19* is seen in ~69% WT either by gain of methylation at the H19-ICR (37%) or by paternal UPD (32%) [28, 29]. Around 10-20% WT patients have constitutional LOI at this locus [30, 31].

**Table 1:** Epigenetics alterations found in Wilms tumours

Aberration	Finding within Wilms tumour
*CASP8* hypermethylation	Frequency of 19% and associated with *RASSF1A* methylation [88]
*GLIPR1/RTVP* hypomethylation	Frequency of 87.5%, results in overexpression [89]
Global hypomethylation	Results in genome instability in tumour cells [90, 91].
Gain of methylation at 6p22.1, 6p21.32 and 11q13.5	Biomarker for WT, can be detected in the circulation of patients [92]
*HACE1* hypermethylation	Frequency of 73% [93]
Hypermethylation of a CTCF binding site downstream of *WT1*	Correlated with high WT1 expression [94]
Hypermethylation of protocadherin cluster at 5q31	Results in expression loss of these proteins at the cell surface [95]
LOI 11p15	Frequency of 69%, results in overexpression of IGF2 and down-regulation of H19 [29, 36, 96]
P16 hypermethylation	Frequency of 23% [97]
*RASSF1* hypermethylation	Frequency of 54% [98]
*WT1*-antisense transcript hypomethylation	Results in biallelic expression [99]

### Evidence that the IGF pathway is disrupted in Wilms tumour

The H19-ICR (which regulates expression of paternally imprinted *IGF2* and maternally imprinted *H19*) contains differentially methylated domains (DMD) and is located between *IGF2* and *H19* [32]. The ICR comprises CTCF (CCCTC-binding factor, zinc finger protein) binding sites and acts by regulating interactions between both gene promoters and their shared enhancers downstream of *H19* [33]. *CTCF* protects the maternal H19-ICR from *de novo* methylation in normal tissue [34]; however aberrant gain of methylation at this allele results in silencing of *H19* expression and transcription of *IGF2* replicating the paternal allele.

Clinically, WT with LOI at 11p15 are associated with perilobar nephrogenic rests (PLNRs), lesions of retained embryonic renal tissue found towards the periphery of the renal lobe, and with blastemal or epithelial-predominant WTs [35, 36] and show increased expression of *IGF2*. Increased expression of *IGF2* results in activation of the insulin signalling pathway. Binding of insulin, IGF1 or IGF2 to the extracellular portion of the insulin receptor (IR), IGF1R or hybrid receptor leads to autophosphorylation of the β-subunit tyrosine kinase, followed by the phosphorylation of additional tyrosine residues. This leads to recruitment of insulin receptor substrates (IRS)1 to IRS4 and other proteins, allowing activation of the PI3K and mitogen activated protein kinase (MAPK) signalling pathways which lead to unregulated protein synthesis, cell cycle progression and cell growth, and prevention of apoptosis [37]. Therefore, in WT, LOI is driving *IGF2* overexpression and oncogenic pathway activation in the cell.

Although constitutional 11p15 abnormalities are found in a small proportion (3%) of patients with sporadic WT (31)and in those with asymmetric overgrowth, including BWS patients, not all develop WT, adding evidence that a second aberrant event is required. Supporting this hypothesis, a strong association between pUPD at 11p15 and *WT1* mutation as well as both mechanisms of LOI at 11p15 and *WTX* mutation are found [29, 30, 36]. Other genes that are commonly mutated in around 30% of sporadic WT cases include *WTX*, *CTNNB1, HACE1, LIN28A, DROSHA* and *FBXW7* although the total number of known mutated genes in WT is limited [38-43].

Further supporting IGF pathway disruption in WT, low level copy number increase of *IGF1R* resulting in aberrant mRNA and protein levels was found in WT, particularly in those with blastemal predominance [44]. Subsequently a mouse model was developed whereby upregulation of *Igf2* was achieved through IGF1R signalling transduced via pIRS1 and pERK1/2. This upregulation alone was insufficient to cause WT, however in combination with *WT1* mutation (present in around 15% WT), 64% of engineered mice developed WT [45]. The resulting WT is probably due to the alteration in normal differentiation of the induced nephrogenic mesenchyme caused by *WT1* mutation together with increased IGF signalling driving proliferation of these cells. This showed that although 11p15 defects are one of the most common aberrations in WT, additional events are necessary for WT development.

Relating this epigenetic aberration to patient treatment, in chemotherapy-naïve tumours, the stage I favourable histology WT weighing less than 550g in children younger than 24 months of age treated with surgery alone showed a significant association between LOH at 11p15, *WT1* mutation and relapse [46]. This finding is being tested in the current COG clinical trial (AREN0532) and if validated, 11p15 methylation analysis may be used to select patients with stage I favourable histology WT who do not require adjuvant therapy [47]. In chemotherapy treated tumours, the relationship between 11p15 disruption and patient outcome remains to be addressed.

**Table 2:** A summary of the agents discussed that target the IGF pathway or epigenome

Mechanism	Compound	Type	Status	Tumour (patients enrolled)
IGF2 inhibitor	m-Cresol	small molecule	developed	not tested *in vivo*
	Myristic acid	small molecule	developed	not tested *in vivo*
IGF1R inhibitor	BMS-754807	ATP-competitive small molecule	developed	xenograft models
	IMC-A12	monoclonal antibody	phase III	hepatocellular carcinoma (n=24)
	NVP-AEW541	small molecule inhibitor	pre-clinical	Ewing sarcoma and neuroblastoma
	Figitumumab (CP-751,871)	monoclonal antibody	phases II/III	Ewing's sarcoma (n=138)
	R1507	monoclonal antibody	phases I/II	Ewing's sarcoma and thymoma (n=29)
	AMG-479	monoclonal antibody	phases III	pancreatic carcinoma (n=800)
	gemcitabine	monoclonal antibody	phases III	pancreatic carcinoma (n=800)
	ganitumab	monoclonal antibody	phases III	pancreatic carcinoma (n=800)
	figitumumab	monoclonal antibody	phases I	myeloma (n=47)
	dalotuzumab	monoclonal antibody	phase I	paediatric solid tumours (n=21)
	cixutumumab	monoclonal antibody	phases II	refractory solid tumours (n=10 patients with WT)
	linsitinib	small molecule inhibitor	phases II/III	non-small Cell Lung Cancer (n=205), ovarian cancer (n=79), adrenocortical carcinoma (n=139)
DNA methyltransferase inhibitors	5-azacytidine	analogue of cytidine	FDA approved for use	myelodysplastic syndrome, acute myeloid leukemia
	5-aza-2′-deoxycytidine	analogue of cytidine	FDA approved for use	myelodysplastic syndrome, acute myeloid leukemia
	MG98	anti-sense nucleotides	phase I and II, terminated	metastatic renal cancer (n=17)
	Zebularine	analog of cytidine	developed	acute lymphoblastic leukemia
	GSK126	small-molecule inhibitor of EZH2 methyltransferase activity	pre-clinical	EZH2 mutated B-cell lymphoma and follicular lymphoma
histone remodelling inhibitors	suberanilohydroxamic acid	histone deacetylase inhibitors	FDA approved for use	cutaneous T-cell lymphoma
	depsipeptide	amino acid-containing small molecule or chain	FDA approved for use	cutaneous T-cell lymphoma
	Romidepsin	histone deacetylase inhibitors	FDA approved for use	cutaneous T-cell lymphoma

### Potential for IGF2 pathway inhibition

Having establishing that the majority of WT, independent of tumour histology [29, 36], are dependent on the IGF signalling pathway it follows that treatment strategies aimed at its inhibition may be useful in the clinic. Currently there are no clinically available drugs which target IGF2 directly although two small molecules are currently under development, m-Cresol and Myristic acid.

**Figure 1:**
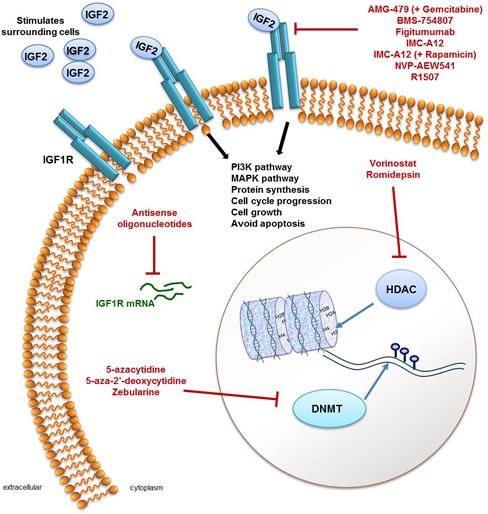
A summary of agents that target either the IGF pathway or the epigenome IGF signalling occurs through stimulation of insulin-like growth factor 1 receptors (IGF1R; shown in blue in the cell membrane) by IGF2 and results in activation of the PI3K and MAPK signalling pathways, an increase in protein synthesis, cell cycle progression, cell growth and avoiding apoptosis. Activation of the IGF pathway through overexpression of IGF2 is frequently seen in Wilms tumour (WT) and is therefore a viable therapeutic target. IGF pathway inhibitors include antisense oligonucleotides which target IGF1R mRNA (green) to prevent their translation into functional protein or molecules that target the IGF1R, which include AMG-479 in combination with Gemcitabine, BMS-754807, Figitumumab, IMC-A12 (alone or with Gemcitabine), NVP-AEW541 and R1507. As frequent epigenomic aberrations are also found in WT (see main text and Table 1) targeting enzymes that regulate DNA methylation (DNA methyal transferases; DNMT) or that regulate histone modifications (histone deacetylases; HDAC) may be a viable therapy for patients with WT. In terms of targeting the epigenome, 5-azacytidine, 5-aza-2'-deoxycytidine and Zebularine are DNMT inhibitors and Vorinostat and Romidepsin are both HDAC inhibitors, preventing normal enzymatic activity.

Instead, the IGF2 receptor, IGF1R, seems to be the most viable therapeutic target due to its redundancy in normal growth and its role in tumorigenesis and growth in cancer [48]. Approaches to targeting *IGF1R* activity include impeding expression, blocking IGF1R interaction with its ligands or preventing receptor activation (Figure 1). There is also novel evidence suggesting that reduction in dietary protein intake may inhibit tumour growth possibly through the inhibition of the IGF/AKT/mTOR pathway [49].

Impeding expression involves targeting *IGF1R* mRNA using antisense oligonucleotides complementary to the translational start site. Expression of such oligonucleotides in glioblastoma cell lines inhibited *IGF1* mediated growth in culture [50] and showed a pronounced growth-blocking effect in murine and human cancer cell lines [51, 52]. In Ewing's sarcoma cell lines, after oligonucleotide delivery by plasmid transfection, an increased response due to an increased sensitivity to Doxorubicin was observed, suggesting a potential use in combination therapy [53].

An alternative approach aims to block the interaction between *IGF1R* and its ligands either with small molecule inhibitors or using monoclonal antibodies. There are several fully humanised anti-IGF1R monoclonal antibodies and small molecule inhibitors of *IGF1R* in various stages of clinical development, including in the Pediatric Preclinical Testing Program (PPTP). The IGF1R inhibitor BMS-754807 is an ATP-competitive small molecule that was tested *in vivo* using xenograft models of several paediatric tumours (including WT) and demonstrated significant growth delay [54]. The anti-IGF1R monoclonal antibody IMC-A12 was also used to treat the same WT models but showed very limited efficacy as a single agent [55]. The data is insufficient to draw firm conclusions as neither study evaluated *IGF1R* network status in the cells. In WT cell lines growing in the kidney environment, the use of the *IGF1R* inhibitor NVP-AEW541 resulted in growth inhibition associated with down-regulation of PI3K and MAPK pathways and down-regulation of cell cycle control genes *CCNA2* and *CCNB1*, with drug efficacy dependent on the levels of phosphorylated *IGF1R* [56]. Two anti-IGF1R antibodies, Figitumumab (CP-751,871) and R1507, have shown success in patients with Ewing's sarcoma and thymoma but have not yet been tested in WT patients or animal models [57, 58]. As well as its role in the IGF pathway as a tyrosine kinase, evidence shows that IGF1R also has kinase-independent activity suggesting that combination targeting using both antibodies that target the receptor as well as small molecule inhibitors may be more effective [59].

The lack of response in some *IGF2*-overexpressing WT may be due to the cancer cells overcoming their dependence on IGF signalling, or that the IGF pathway is not the sole dominant driver, thus suggesting that combination therapies may be more effective. In fact, *in vivo* studies of IGF1R antibody AMG-479 with gemcitabine showed additive inhibitory activity in pancreatic carcinoma [60]. Combined inhibition of the *mTOR* and *IGF1R* signalling pathways is an attractive and biologically rational approach to development of anticancer therapy for sarcomas and other solid tumours. Separately, both pathways appear to be relevant in multiple forms of cancer, and preclinical models suggest synergistic antitumor activity with combined inhibition of these pathways. The PPTP has demonstrated that the combination of IGF1R inhibitor IMC-A12 and mTORC1 signalling inhibitor rapamicin have a superior tumour response compared to either agent alone in several models of paediatric cancer, however testing has not been carried out in the WT model [61]. In human WTs, overexpression of IGF2, through allele loss of 11p15.5 or loss of imprinting, frequently co-exists with *WT1* mutation. The same combination of critical genetic events proved essential for tumour formation in a genetically engineered mouse model [45]. ERK1/2 phosphorylation was upregulated in WTs in both species, suggesting a further target for therapy.

Unfortunately, some drugs that target the IGF pathway, when tested in clinical trials have not given encouraging results. Amgen (Amgen Inc. CA, USA) recently terminated ganitumab (AMC-479 [62]), an antibody against IGF1R, for a late-stage pancreatic cancer trial [63]. Earlier in 2010, Pfizer abandoned figitumumab after trials failed to show clinical efficacy in adult cancers (lung carcinoma and myeloma). Several other drug manufacturers have discontinued their own earlier-stage programs directed at the same target. However, Merck are still developing an IGF1R inhibitor dalotuzumab, with an emphasis on the identification of predictive biomarkers for subgroups that benefit most from this drug and Eli Lilly has an IGF1R-targeted antibody, cixutumumab, in phase 2 trials. COG recently carried out a randomised phase 2 study testing cixutumumab in adolescents and children with refractory solid tumours including ten patients with WT. The drug was well tolerated in patients and prolonged stable disease was seen in 15% patients [64]. Although no response in patients with refractory WT was observed, this was a single-agent trial and drugs that only target IGF1R may be non-efficacious as they fail to target the insulin receptor, another tyrosine kinase receptor that is activated by IGF1 and IGF2 in addition to insulin itself. Following this idea, Astellas Pharma developed a drug, linsitinib which is undergoing phase 2 trials for several tumour types, that targets both receptors. The observed poor response may be due to the complexity of the IGF1R/insulin receptor system and parallel growth and survival pathways. However, IGF1R does remain a valid target for selected tumour types and agents targeting IGF1R are attractive therapeutic targets for WT.

An alternative approach to overcome the potential side effects of inhibiting the whole IGF pathway is the inhibition of specific arms of the pathway important for tumour development. The monoclonal antibody mAb 1/41 targets the metalloproteinase, pregnancy-associated plasma protein-A (*PAPP-A*) resulting in indirect inhibition of IGF signalling [65]. Tumours with increased *PAPP-A* activity will benefit from the use of mAb 1/41 with the advantage of local and indirect targeting of the IGF-IR, rather than global targeting which lacks specificity and is likely to interfere with metabolism [66], however there is no information in the literature about *PAPP-A* activity in WT.

### Therapeutic options based on the WT epigenome

It is now clear that cancer development depends not only on genetic alterations but also on epigenetic changes that convey heritable gene expression patterns critical for neoplastic initiation and progression [67]. However, unlike genetic mutations which are permanent, the discovery that these epigenetic marks can be reversed by compounds targeting aberrant transcription factor/co-activator/co-repressor interactions and histone-modifying activities, suggests the possibility that the epigenome of cancer cells may be manipulated with potential therapeutic benefits [68]. Given the proven dysregulation of epigenetic architecture in WT and the pattern, interactions and clinical associations of molecular events in WT there is clear potential for targeting the epigenome to treat WT. A significant and growing number of pharmaceutical companies have research programs that aim to define proteins that target the epigenome. Drugs currently FDA approved or in the pipeline for approval for this purpose include the DNA methyltrans­ferase inhibitors 5-azacytidine (Vidaza and AZA), 5-aza-2′-deoxycytidine (Decitabine, Dacogen and DAC), as well as two histone remodelling inhibitors: suberanilohydroxamic acid (Vorinostat, Zolinza) and depsipeptide (Romidepsin, Istodax) [69], shown in Figure 1. More epigenetic drugs are entering into pre-clinical development every year [70].

Histone deacetylases (HDACs) are critical in the control of gene expression. Molecules that interfere with histone remodelling, for example histone deacetylase inhibitors (HDACi) are an emerging class of therapeutics with potential as anticancer drugs with promising results in clinical trials [71]. To date, two HDACi have received FDA approval for the treatment of cutaneous T-cell lymphoma: Vorinostat and Romidepsin. Clinical trials assessing the effects of various HDACi on haematological and solid malignancies are currently being conducted [72]. Despite the proven anticancer effects of particular HDACi, a detailed understanding of how HDACi with different pharmacological properties affect biological functions *in vitro* and *in vivo* is still missing [73]. Therefore while drugs that target histone remodelling proteins exist and have been tested in both haematological and solid malignancies [74], the potential efficacy of such inhibitors in WT is unknown and unpredictable as no studies have revealed a particular dependence on HDACs in WTs. In addition, the potential downside of drugs that target global chromatin modifications is their broad effect on fundamental cellular mechanisms. They can cause multiple side effects - anorexia, nausea, vomiting, suppression of white blood cells and thrombocytopenia, and profound fatigue [75].

DNA methyl transferase (DNMT) 1, 3a and 3b are enzymes that transfer a methyl group to cytosine residues in DNA. DNMT1 maintains methylation during DNA synthesis whereas 3a and 3b act during embryonic development [76, 77]. A number of different types of DNMT inhibitors are known including non-nucleoside analogues which are antisense oligonucleotides targeting the DNMT family of enzymes, resulting in DNA demethylation. One such inhibitor underwent phase I and phase II trials for treatment of renal cancer but failed due to a lack of efficacy [78].

The more common inhibitors for DNMT are nucleoside analogues that become phosphorylated and are incorporated into DNA during replication also resulting in global DNA demethylation. The DNMT inhibitors Vidaza and Decitabine are the most successful epigenetic drugs to date and are still the most widely used as epigenetic modulators, even though their application for oncological diseases is restricted by their relative toxicity and poor chemical stability. Decitabine was approved for the treatment of myelodysplastic syndrome (MDS) in 2006 and shows anti-leukemic activity against acute myeloid leukemia (AML). Its clinical activity against solid tumours is under investigation, but initial studies in patients with solid tumours have demonstrated only limited responses. However, as there is a close correlation between the concentration of Decitabine, exposure time, demethylating effects and thus anti-tumour activity, these patients potentially received a suboptimal exposure to the drug compared to that expected from *in vitro* and *in vivo* animal studies [79]. Zebularine (1-(β-D-ribofuranosyl)-1,2-dihydropyrimidin-2-one), a more stable and less toxic cytidine analog, is another DNMT inhibitor which also shows inhibitory activity towards cytidine deaminase [80]. Zebularine preferentially targets cancer cells and exhibits low toxicity toward normal cells and mice [81, 82]. Zebularine induces apoptosis and decreases clonogenic capacity of acute lymphoblastic leukemia cell lines in a dose-dependent manner [83]. Although it is orally available and more stable than FDA-approved Vidaza and Decitabine, its clinical benefit is yet to be evaluated.

As WT shows a triphasic morphology, demethylating agents may show different activity in each cell type however, as no evidence currently exists as to whether one cell type may respond better, this would also need to be assessed.

Finally, enhanced expression of EZH2 in WT, a key polycomb group protein that functions to repress transcription through critical histone 3 methylation markers, has been identified to correlate with WT progression [84]. Agents targeting EZH2 such as GSK126 have entered pre-clinical and clinical development [85, 86].

## CONCLUSIONS AND CHALLENGES

Here, we have discussed a wide range of agents that target the IGF signalling pathway or the epigenome (summarised in Table 2). It is clear that epigenetic alterations play a key role in a significant fraction of WTs and result in LOI at *H19*/*IGF2* [33]. This epimutation has also been observed in the associated nephrogenic rests (precursor lesions) and surrounding normal kidney of patients with WT. Furthermore, other epigenetic aberrations have been found within the tumour tissue and it appears that the founding event in a large subset of WTs is the failure to complete nephrogenesis, thus leaving lesions with an epigenetically unstable composition.

Considering this evidence, WT seems at present to be an ideal model for epigenetic therapy. While global demethylators can be assessed for use as adjuvant therapy, most of the epigenetic drugs under development require effective cell proliferation, suggesting that they may only be effective in a subset of tumours.

We have discussed the use of targeting IGF signalling in WT as a possible therapeutic strategy. Most importantly, the assessment of IGF signalling disruption in clinical trials may help to identify the patient subgroup most likely to benefit from treatment with pathway inhibitors. Moreover, the COG study AREN0532, which is currently analysing the relationship between 11p15 methylation in very low risk WT and patient outcome, may support previous evidence that patients with 11p15 LOH are more likely to relapse which would aid future patient stratification and treatment decision planning. 11p15 disruption and its association with outcome remains to be addressed in pre-treated tumours.

At present, targeting IGF signalling has been tested on WT cell lines and xenograft models which showed significant growth delay. Given *IGF2* overexpression in WT, targeting IGF1R seems plausible. However, it must be considered that tumours can overcome IGF pathway dependence and therefore combination therapy might be a more effective option, though the effective pathways to be co-inhibited have not yet been defined. Furthermore, the use of new epigenetic therapies needs to be established before we can consider assessing efficacy in clinical trials. Should this therapeutic approach be considered in the future, the status of 11p15 imprinting would have to be assessed at diagnosis to determine whether IGF-targeted therapy would be beneficial for a patient, perhaps in the same way that assessing ERBB2 status impacted on breast cancer treatment. This can be done by methylation-specific MLPA (MS-MLPA), a simple method based on digestion of genomic DNA-probe hybrid complexes combined with methylation-sensitive endonucleases. As a quantitative method, MS-MLPA discriminates between methylation of one, both or none of the alleles making it a powerful screening tool [87]. Further clinical trials assessing IGF1R inhibitors *in vivo* as well as mRNA therapy blocking *IGF1R* expression are potential avenues for further consideration.
